# Frequent detection of *‘azole’* resistant *Candida* species among late presenting AIDS patients in northwest Ethiopia

**DOI:** 10.1186/1471-2334-13-82

**Published:** 2013-02-12

**Authors:** Andargachew Mulu, Afework Kassu, Belay Anagaw, Beyene Moges, Aschalew Gelaw, Martha Alemayehu, Yeshambel Belyhun, Fantahun Biadglegne, Zewdu Hurissa, Feleke Moges, Emiko Isogai

**Affiliations:** 1 Department of Microbiology, Parasitology and Immunology, College of Medicine and Health Sciences, University of Gondar, Gondar, Ethiopia; 2 Institute of Virology, Faculty of Medicine, University of Leipzig, Johannisallee 30, 04103, Leipzig, Germany; 3 Department of Internal Medicine, College of Medicine and Health Sciences, University of Gondar, Gondar, Ethiopia; 4 Department of Disease Control and Molecular Epidemiology, Health Sciences University of Hokkaido, Ishikari-Tobetsu, Hokkaido, Japan

**Keywords:** *Candida* species diversity, *In vitro* susceptibility, Late presenters, Northwest Ethiopia

## Abstract

**Background:**

The chronic use of antifungal agents in the treatment of fungal infection in general and oropharyngeal candidiasis mainly in AIDS patient’s leads to the selection of strain resistant to these therapies and a shift in the spectrum of *Candida* species. This study determines the species diversity and *in vitro* susceptibility of *Candida* isolates from late presenting AIDS patients in northwest Ethiopia.

**Methods:**

Two hundred and twenty one HIV/AIDS patients were assessed with a standardized evaluation form at enrolment. Oral rinses were cultured on CHROMagar plates at 37°C for 48 hours and *Candida* species identification were made following standard microbiological techniques. *In vitro* drug susceptibility tests were made using broth microdilution method.

**Results:**

The colonization rate of *Candida* species was found to be 82.3% (177/215). *C. albicans* was the predominant species isolated from 139 (81%) patients but there was a diversity of other species. *C. glabrata was* the most frequent non-*albicans* species isolated in 22.5% (40/177) of the patients followed by *C. tropicalis* 14.1% (27/177), *C. krusei* 5.6% (10) and other unidentifiable *Candida* species 4% (7/177). Recurrent episodes of oropharyngeal candidiasis and previous exposure to antifungal drugs were found to be predisposing factors for colonization by non-*albicans* species. Irrespective of the *Candida* species identified 12.2% (11/90), 7.7% (7/90) and 4.7% (4) of the isolates were resistant to fluconazole, ketoconazole and itraconazole, respectively. In contrast, resistance to micafungin, amphotericin B and 5-Fluorocytosine was infrequent.

**Conclusion:**

HIV/AIDS patients are orally colonized by single or multiple albicans and non- albicans *Candida* species that are frequently resistant to azoles and occasionally to amphotericin B, 5-Fluorocytosine and micafungin. These highlight the need for national surveillance for examining *Candida* epidemiology and resistance to antifungal drugs.

## Background

*Candida* species are among the gut flora, the many organisms which live in the human mouth and gastrointestinal tract. Up to 75% of healthy individuals carry the yeast *Candida* as part of their normal commensal oral microflora. However, in the past 3 decades, infections due to *Candida* species have increased dramatically and are of particular importance because of the rising number of immunocompromised patients [1]. Oropharyngeal candidiasis (OPC) is one of Candidal infection which continues to be a common opportunistic infection in patients infected by the human immunodeficiency virus (HIV). It increase morbidity and mortality and reduces length and quality of life of HIV/AIDS patients. It occurs in up to 90% of the patients at some point during the course of HIV disease and is associated with high viral loads, low CD4^+^ T cell count (< 200 cells/mm^3^) and disease progression [1-4]. The prolonged course of HIV infection predisposes these patients to recurrent episodes of OPC that can increase in frequency and severity with progressive HIV disease [5]. Today although, the introduction of highly active antiretroviral therapy (HAART) has dramatically reduced the incidence of opportunistic infections among HIV-positive people who have received the drugs, OPC with a shift in the spectrum of *Candida* species remains the most frequent HIV-associated oral lesion in most developing countries, including Ethiopia [2,5], where access to HAART is still limited.

The high incidence of mucosal and deep seated forms of candidiasis has resulted in the use of systemic antifungal agents, especially fluconazole and itraconazole [6]. The widespread these antifungal agents have been followed by an increase in antifungal resistance and by a noticeable shift toward non albicans species with relative resistance to fluconazole and itraconazole [7] and there have been reports of emergence of resistance to antifungal agents in HIV/AIDS patients with OPC [8-13]. Certain non-*albicans* species, for example *C. glabrata* and *C. krusei* are inherently less susceptible to fluconazole than *C. albicans* and have been isolated with increasing frequency in HIV-infected patients [14]. The resistance of *Candida* isolates to currently available antifungal drugs is a highly relevant factor because it causes important implications for morbidity and mortality.

Like other African countries, the present guideline of the Ethiopian Ministry of Health (MOH) for the management of candidiasis include fluconazole, as a first choice drug and ketoconazole, micoconzole ointment as alternative antifungal agents. However, clotrimazole oral torches, nystain oral suspension, genital violet solution and 2% sodium benzoate solution are also prescribed in routine clinical practice [15,16]. In Ethiopia, fungal culture and *in vitro* antifungal testing are not performed in routine clinical laboratory practice and data on the circulating *Candida* species and antifungal drug resistance are inadequate except for a single study conducted over a decade [13]. Recently, we have reported *Candida* species diversity and found fluconazole resistance strains of *Candida* species among 13 AIDS patients with advanced diseases from central Ethiopia [9,17]. These initiated us to conduct a large scale study in tropical settings of northwest Ethiopia where HIV/AIDS patients present late in the course of the diseases and OPC is observed as a major AIDS defining event. The objective of this study was therefore, to determine the diversity of *Candida* species and *in vitro* susceptibility of *Candida* species isolated from AIDS patients to six common antifungal agents.

## Methods

### Settings

Ethiopia is a low income country with an estimated HIV prevalence of 2.1% in 2009 [18] and where OPC remains the most frequent HIV associated oral lesion. Gondar University teaching hospital is a tertiary level referral and teaching hospital in northwest Ethiopia rendering health service for over 5 million inhabitants. Northwest Ethiopia, the area where the study was conducted, is a high HIV prevalence region with almost all HIV infections occurs through the heterosexual route. The Gondar University teaching hospital HIV clinic consists of an Integrated Counselling and Testing Centre, in addition to a CD4^+^ T cells monitoring laboratory and ART pharmacy. HIV treatment and monitoring is free of cost.

### Patients

Two hundred and twenty one consecutive patients were recruited at the University of Gondar teaching Hospital HIV clinic from April 2008-November 2008 when they were referred to the clinic from voluntary HIV counselling and testing (VCT) centre and/or from out patients departments (OPD) and/or other health institutions. Patients were eligible for the study if they were HIV-positive, greater than 18 years of age and willing to participate in the study. All the patients were antiretroviral naïve with various WHO clinical stages. Patients were assessed with a standardized evaluation form at enrolment where sociodemographic data and clinical findings were recorded. Patients were interviewed to determine the previous episodes of OPC and use of antifungal agents. Primary OPC was defined as the one that occur for the first time and recurrent OPC as the second episode and above during the course of HIV disease. Apparently healthy HIV negative blood donors (free from blood borne infection, chronic illness and acute infections) were also included and served as a control.

### CD4^+^ T cell counts

Blood was collected in EDTA tubes from each patient for enumeration of CD4^+^ and CD8^+^ T cells using a fluorescent activated cell sorter (FACS) count machine (Becton Dickinson, San Jose, California, USA) following the manufacturer’s protocol. According to the first CD4^+^ T cells count before HAART, patients were classified as early presenter (CD4^+^ T cells > 200 cells/μl) and late presenter (CD4^+^ T cells <200 cell/μl). The Federal HIV/AIDS treatment guideline do not include a formal definition of late presentation, but has been strongly recommend that any patients with CD4^+^ T cells count of < 200 cells/μl irrespective of WHO clinical stage should receive treatment during the study period [16].

### Isolation and identification of Candida species

After informed and written consent was obtained, the study subjects were instructed to provide rinse of oral cavity as we have previously described elsewhere [9,17]. Briefly, oral rinses were obtained from each subject by asking them to rinse their mouth with 5 ml of sterile normal saline (0.85% NaCl solution) for 30 seconds and to expectorate the rinse into a sterile container. The mixture was then thoroughly mixed for homogenization and 0.1 ml of the fluid was then, plated to a CHROMagar plate (CHROMagar, Paris, France), a selective medium for isolation of *Candida* species which was prepared as per the manufacturer’s instruction. After 60 seconds, the plates were drained and incubated at 37°C for 48 hours. Presumptive identification of *Candida* was made based on color of the colonies as per the protocol of the manufacturer as *C. albicans*, *C. tropicalis* and *C. glabrata*. Colonies were counted irrespective of single or mixed colonies. A cut off point was established for colony counts, so that 400 or greater CFU/ml was considered a high CFU count as previously reported [18]. In 25% of the isolates, further identification of *Candida* was done using Tobaco agar which we have previously evaluated using PCR as gold standard [17]. The isolates were also confirmed by API 20C AUX identification System for yeasts (BioMerieux, Marcy L’Etoile, France). Isolates were stored at −40°C with 50% glycerol based Tryptose Soya Broth supplemented with 0.5% yeast extract and cultured on CHROMagar at 37°C prior to being tested for their susceptibility.

### In vitro antifungal susceptibility test

The *in vitro* activities of fluconazole, ketoconazole, 5-Fluorocytosine and amphotericin B (Sigma Aldrich, Steinheim, Germany), itraconazole (LKT Laboratories, Inc US) and micafungin (Astellas Pharma, US) were assessed according to CLSI [19] with a slight modification. Briefly, inoculums suspension was adjusted to a 0.5 McFarland Standard to produce 1 × 10^6^ to 5 × 10^6^ cells per ml. A working suspension was made as by a 1: 100 dilutions. Immediately, 10 μl of this saline suspension was transferred to 10 ml of sterile saline. After the suspension was mixed, it was poured into an inoculation tray. By use of a 12-channel pipette and sterile tips, 100 μl of the inoculum was added to wells 1 to 11 (wells 1 to 10 contained 100 μl a serial twofold dilutions of antifungal agents; well 11 was the growth control without the antifungal); well 12 was the sterility control or blank) in Tryptose Soya Broth (TSB) a non synthetic medium supplemented with 0.5% yeast extract. TSB contains Casein and Soybean Meal-nitrogen sources; Dextrose-carbon energy source that facilitates organism growth; Sodium Chloride-maintains osmotic balance; Dipotassium Phosphate-a buffering agent and provides an optimal recovery of *C. albicans* and *C. glabrata*[20].

The range of fluconazole concentrations tested was 0.125–64 μg/ml and for itraconazole, ketoconazol, 5-Fuorocytosine and micafungin the range was 0.016–16 μg/ml and for amphotericin B the range was 0.125–8 μg/ml. This inoculum is equivalent to 5.0 × 10^2^ to 2.5 × 10^3^ cells per ml. The microtiter plate was shaken on a plate shaker for 30 seconds to ensure even distribution of the inoculums. After incubation at 37°C for 48 hours, the microtiter plate was taken out from the incubator and shaken for 5 minutes. The minimum inhibitory concentrations (MICs) were determined by reading the optical density (OD) of each well at 530 nm spectrophotometrically. The ODs of the blanks were subtracted from the ODs of the inoculated plates and the percentages of growth for each well were calculated. The MICs of the azoles (fluconazole, itraconazole, ketoconazole) were defined as the lowest concentrations that inhibit growth by 50%. Tests were done in duplicate and the MIC values were the average of the duplicate measurement. Resistance breakpoint definitions were based on CLSI guideline [19] and are as follows for azoles: Fluconazole: resistant ≥ 64 μg/ml, susceptible-dose dependent (S-DD) 16-32 μg/ml and susceptible ≤ 8 μg/ml; itraconazole: resistant ≥ 1 μg/ml, SDD 0.25-0.5 μg/ml and susceptible ≤ 0.125 μg/ml; ketoconazole: resistant ≥ 4 μg/ml. Resistance breakpoint definitions for the fluorinated pyrimidine analogue (5-Fluorocytosine) were also as follows: susceptible ≤4, SDD 8–16 and resistant >16 μg/ml μg/ml. There were no CLSI endpoints defined for the polyene (amphotericin B) and their MICs were defined as the lowest concentrations that inhibit growth by 100%. However, in this study we used the values of > 2 μg/ml for resistance to amphotericin B as endpoints as reported by Brito *et al.*[21]. Resistance for the echinocandin antifungal agent (Micafungin) were defined as the lowest concentrations that inhibit growth by 50% [19]. Standard strains of *C. albicans* ATCC 10231 were used for quality control. MIC_50_ and MIC_90_ are the minimum inhibitory concentration values that able to inhibit 50% and 90% of the organism, respectively.

### Statistical analysis

The data was computerized in Microsoft Excel and transferred to SPSS version 17 for analysis. Differences between continuous variables were assessed by means of two-tailed Student’s t-test for differences in means and percentages. Differences between categorical variables were evaluated by chi square test. P values < 0.05 were considered statistically significant.

### Ethical issues

This study was approved by the University of Gondar Institutional Review Committee. After the goals and objectives of the study were explained to the study participants, informed consent was obtained from each subject.

## Results

### Patient’s characteristics

Two hundred and twenty one HIV-1 infected patients (92 males and 128 females) were included in the study. The mean ± SD age was 32.5 ± 8.5 years (range 16–50 years). The demographic characteristics and baseline clinical and immunological data of the patients is summarized in Table 1. The median CD4^+^ T cells count among males (207 cells/μl) and females (213 cells/μl) was not statistically different. One hundred and twenty five (56.7%) of the patients were found to be late presenters and the rest 96 (43.3%) were early presenters. Frequency of late presentation was highest in females and age group >40 years, with being statistically significant (p < 0.001). More than 65% of these patients were with advanced AIDS diseases (WHO clinical stages 3 and 4). In a multivaraint logistic regression analysis, we found that females were at a significantly higher risk to present late compared to their male counterparts (late presentation: OR 0.49; 95% CI 0.41 – 0.54). Upon oral examination, 82 (37.5%) had clinical oral cadidiasisis with pseudomembraneous candidiasis (43), erythematous candidiasis (33), *candida* leucoplakia (3) and angular chielatis (3) types. Of the 82 patients with clinical OPC, 11 patients were early presenters with CD4^+^ T cell count of >200 cells/μl.

**Table 1:** Demographic and baseline clinical and immunological characteristics of the patients, northwest Ethiopia, 2008

**Variables**	**No (%)**
Sex	
Male	93 (42.0)
Female	128 (58.0)
Age (in years)	
0–19	3 (1.4)
20–29	87 (39.4)
30–39	80 (36.2)
40–49	39 (17.6)
≥50	12 (5.4)
CD4^+^ T cell count (cells/mm3)	
0–49	33 (15.3)
50–99	29 (13.5)
100–199	57 (26.5)
200–349	55 (25.6)
>350	41 (19.1)
WHO clinical stages	
I	30 (13.6)
II	45 (20.4)
III	138 (62.2)
IV	8 (3.6)
Malnutrition (BMI in kg/m^2^)	
Sever (<15.9)	26 (12.3)
Moderate (16–16.9)	24 (11.4)
Mild (17–18.4)	49 (23.2)
Normal (>18.5)	112 (53.1)

### Candida species diversity

Of the total 221 patients enrolled in the study, it was possible to collect oral rinse from 215 patients. The colonization rate of *Candida* species was found to be 82.3% (177/215). As expected *C. albicans* was the most predominate isolate occurring in 78.5% (139/177) of the patients either as the single species (99 subjects) or mixed with other *Candida* species (40 subjects). *C. glabrata was* the most frequent non-*albicans* species isolated in 22.5% (40/177) of the patients followed by *C. tropicalis* 14.1% (27/177), *C. krusei* 5.6% (10) and other unidentifiable *Candida* species 4% (7/177). Multiple *Candida* species colonization was observed in 42 (23.7%) patients (Table 2) and the over all species specific isolation was 136, 37, 25, and 10 for *C. albicans, C. glabrata, C. tropicalis* and *C. krusei*, respectively out of the total 215 oral rinse samples. Three forth of the patients with multiple *Candida* colonization had reported a recurrent episode of OPC and half of them had previous exposure to antifungal agents. *Candida* colonization rate was not dependent on age, CD4^+^ T cell count and WHO clinical stage. The majority (72%) of the patients with clinical OPC were colonized by *C. albicans* and the rest by non-albican *Candida* species. Among the 36 patients who have been colonized by non *Candida* species alone, the majority (78%) had exposure to common antifungal agents and had reported previous episodes of OPC. Limited Candida species diversity was observed among healthy controls (*C. albicans, C. glabrata and C. tropicalis*) compared with AIDS patients (*C. albicans*, *C. glabrata,* C*. tropicalis*, *C. krusei, C. parapsilosis, and C. kefyr)*.

**Table 2:** Distribution of oral Candida isolates among AIDS patients, northwest Ethiopia, 2008

**Species**	**No (%)**	**Total isolates**
*Candida albicans*	99 (46.0)	137
*Candida tropicalis*	15 (7.0)	40
*Candida glabrata*	12 (5.6)	25
*Candida krusei*	2 (0.009)	10
*Candida albicans + Candida glabrata*	22(10.2)	-
*Candida albicans + Candida tropicalis*	7(3.2*)*	-
*Candida albicans + Candida krusei*	7(3.2)	-
*Candida tropicalis + Candida glabrata*	2(0.009)	-
*C. albicans* + *C. tropicalis* + *C. glabrata*	1	-
*C. albicans + C. krusei* + *C. tropicalis* + *C. glabrata*	1	-
Other Candida species*	7 (3.2)	7

*: Unidentified Candida species.

### In vitro antifungal drug susceptibility

*In vitro* antifungal drug susceptibility test was done on randomly selected isolates of *C. albicans* (25), *C. glabrata* (25) and unidentified *Candida* species (5) and for all isolates of *C. tropicalis* (25) and *C. krusei* (10). As shown in Table 3, irrespective of the *Candida* species identified 12.2% (11/90), 3.3% (3/90) and 84.4% (76/90) of the isolates were resistant, SDD and susceptible to fluconazole. The MICs value of susceptible *Candida* isolates (irrespective of the species) to fluconazole was 7.32 μg/ml with 6.9 μg/ml, 6.1 μg/ml, 8.24 μg/ml and 8.04 μg/ml for C*. albicans, C. tropicalis*, *C. glabrata* and *C. krusei*, respectively. Seven of the isolates (7.7%) were found to be resistant for ketoconazole with a mean ± SD MIC value of 4.92 ± 2.4. Four (4.7%) of the isolates were resistant to itraconazole, while 3 (3.3%) were SDD and the remaining 83 (92.2%) of the isolates were found to be susceptible to itraconazole. Three (3.5%), 2 (2.3%) and 2 (2.3%) of all the isolates were found to be resistant to amphotericin B, 5-Fluorocytosine and micafungin, with relatively low MIC value of 0.13, 1.26 and 1.28 μg/ml, respectively. Of the 90 *Candida* isolates tested for *in vitro* susceptibility, 9 (10.5), 8 (9.4), 6 (7.1), 2 (2.3), 3 (3.5) and 3 (3.5) of the isolates were found to be resistance for fluconazole, ketoconazole, itraconazole, micafungin, amphotericin B and 5- Fluorocytosine, respectively (Table 3). Multidrug resistance (resistance to two or more antifungal) strains were also detected in 8 isolates (3 *C. albicans, 2 C. glabrata,* 1 *C. tropicalis,* 1 *C. krusei* and 1 other *Candida* species). Thirty one (36%), 5 (5.8%), 2 (2.3%) and 3 (3.5%) of the isolated *Candida* species were found to be resistant to one, two, three and four of the antifungal agents tested, respectively. Four (16%), 3 (12%) and 18 (72%) of *C. albicans* isolates were found to be resistant, SDD and susceptible to fluconazole, respectively. Only two of the isolated *C. glabrata* isolates (8%) was resistant to fluconazole, while 22 (88%) were susceptible and 1 (4%) were SDD. Similar pattern of susceptibility and resistance was also observed to this agent by *C. tropicalis*. All except one *C. krusei* isolates were susceptible to fluconazole. The susceptibility/resistance profile of the *Candida* isolates to other antifungal agents is summarized in Table 4. Moreover, a total of 20 *Candida* isolates (10 *C. albicans,* 5 *C. glabrata* and 5 *C. tropicalis)* obtained from HIV negative individuals were also evaluated for antifungal drug susceptibility. A significant difference on MIC value was observed on all isolates from healthy controls when compared with MIC value of *Candida* species isolated from AIDS patients (data not shown).

**Table 3:** **Antifungal susceptibility profile of oral *Candida *isolates from AIDS patients, northwest Ethiopia, 2008/09**

**Species/Antifungal agent**	**MIC (μ/ml)**			**SDD**	**R: n (%)**
	**MIC Range**	**MIC**_**50**_	**MIC**_**90**_		
All isolates (90)					
Fluconazole	0.125–64	0.25	2	3 (3.3)	11 (12.2)
Ketoconazole	0.016–16	2	4	-	7 (7.7)
Itraconazole	0.016–16	0.016	0.063	3 (3.3)	4 (4.7)
Amphotericin B	0.125– 8	0.125	0.125	-	3 (3.5)
5-Fuorocytosine	0.125–16	0.125	0.125	-	2 (2.3)
Micafungin	0.015–8	0.06	1.2	-	2 (2.3)
*C. albicans* (25)					
Fluconazole	0.125–64	0.25	2	1 (4)	4 (16)
Ketoconazole	0.016–16	-	3 (12)		
Itraconazole	0.016–16	0.016	0.063	3 (12)	2 (8)
Amphotericin B	0.125–8	0.5	0.5	-	1 (4)
5-Fuorocytosine	0.125–16	-	-	-	1 (4)
Micafungin	0.015–8	-	-	-	1 (4)
*C. glabrata* (25)					
Fluconazole	0.125–64	0.25	2	1 (6)	3 (8)
Ketoconazole	0.016–16	-	-	-	1 (4)
Itraconazole	0.016–16	0.016	0.063	2 (12)	1 (4)
Amphotericin B	0.125–8	0.5	-	-	-
5-Fuorocytosine	0.125–16	-	-	-	1 (4)
Micafungin	0.015–8	-	-	-	-
*C. tropicalis* (25)					
Fluconazole	0.125–64	0.25	2	-	2 (8)
Ketoconazole	0.016–16	-	1 (4)		
Itraconazole	0.016–16	0.016	0.063	2 (12)	1 (4)
Amphotericin B	0.125–8	0.5	0.5	-	-
5-Fuorocytosine	0.125–16	-	-	-	1 (4)
Micafungin	0.015–8	-	-	-	-
*C. krusei* (10)					
Fluconazole	0.125–64	0.25	2	1 (10)	1 (10)
Ketoconazole	0.016–16	-	-	-	1 (10)
Itraconazole	0.016–16	0.016	0.063	1 (10)	-
Amphotericin B	0.125–8	0.5	-	-	-
5-Fuorocytosine	0.125–16	-	-	-	-
Micafungin	0.015–8	-	-	-	-
Other Candida species* (5)					
Fluconazole	0.125–64	0.25	2	-	1 (20)
Ketoconazole	0.016–16	-	-	-	1 (20)
Itraconazole	0.016–16	0.016	0.063	1 (10)	-
Amphotericin B	0.125–8	-	-	-	-
5-Fuorocytosine	0.125–16	-	-	-	-
Micafungin	0.015–8	-	-	-	-

*: Unidentified Candida species.

**Table 4:** Multi drug resistance profile of Candida isolates from oral cavity of AIDS patients, northwest Ethiopia, 2008

**Candida species**	**Pattern**	**Antifungal agents**						**MDR**
		**Fluconazole**	**Ketoconazole**	**Itraconazole**	**Amphotericin B**	**5-Fluorocytosine**	**Micafungin**	
*C. albicans* (25)	S	20 (80)	22 (88)	20 (80)	24 (96)	24 (96)	24 (96)	-
	SDD	1 (4)	-	3 (12)	-	-	-	-
	R	4 (16)	3 (12)	2 (8)	1 (4)	1 (4)	1 (4)	3 (12)
*C. glabrata* (25)	S	22 (88)	24 (96)	22 (88)	25 (100)	24 (96)	25 (100)	-
	SDD	1 (4)	-	2 (8)	-	-	-	-
	R	3 (12)	1 (4)	1 (4)	-	1 (4)	-	2 (8)
*C. tropicalis* (25)	S	23 (92)	24 (96)	22 (88)	25 (100)	24 (96)	25 (100)	-
	SDD	-	-	2 (8)	-	-	-	-
	R	2 (8)	1 (4)	1 (4)	-	1 (4)	-	2 (8)
*C. krusei* (10)	S	8 (80)	9 (9)	9 (90)	10 (100)	10 (100)	10 (100)	-
	SDD	1 (10)	-	1 (10)	-	-	-	-
	R	1 (10)	1 (10)	-	-	-	-	1 (10)
Other Candida spp* (5) S	4 (80)	4 (80)	4 (80)	-	-	-	-	
	SDD	-	-	1 (20)	-	-	-	-
	R	1 (20)	1 (20)	-	-	-	-	1 (20)

Keys: S-Susceptible; SDD-Susceptible dose dependent; R-Resistant; MDR-Multi drug resistance; *: Unidentified Candida species.

### Cross resistance

From the total of 9 *Candida* isolates which were resistant to fluconazole, 2 and 5 were concurrently found to be resistant to ketoconazole and itraconazole, respectively. Three of the ketoconazole resistant isolates were found to be resistant to fluconazole. There was a significant difference in frequency of resistance to fluconazole between the ketoconazole resistant and susceptible *Candida* isolates (p < 0.05). Two ketoconazole resistant strains were resistant to 5-Fluorocytosine. None of azole resistant isolates was found to be resistant to micafungin and amphotericin B.

### Risk factors for Candida load and drug resistance

There was no difference on median *Candida* counts in men (30 CFU/ml; range, 0 to 48,000 CFU/ml) and women (600 CFU/ml; range, 0 to 82,000 CFU/ml; *p* = 0.09) and in age groups. However, there was a significant relationship between CD4^+^ T cell count and *Candida* CFU counts (*p* = 0.0021). Patients with clinical signs of candidiasis had significantly (*p* < 0.05) higher *Candida* counts (12, 346 CFU/ml) compared with patients without clinical candidiasis (6783 CFU/ml). On the other hand, there was no significant difference on *Candida* count among patients with and without drug resistant *Candida* strains (9769 vs. 10, 341 CFU/ml). However, patients colonized by *C. albicans* had higher CFU than patients colonized by non-*albicans* (*p* = 0.03) and multiple colonization was associated with higher CFU counts (*p* = 0.001). The difference on Candida load was significantly low on all isolates from healthy controls when compared with the Candida load of *Candida* species isolated from AIDS patients (2363 CFU/ml vs 67, 781 CFU/ml; *p* = 0.0001).

Attempt was made to determine the number of episodes of OPC and use of antifungal agents. Thirty seven patients had reported as the second episode of OPC. Forty three (19.5%) of these patients had previous exposure for either for systemic (fluconazole) or topical (clotrimaxzole and/ or ketoconazole) antifungal agents. The mean duration of the antifungal therapy and sampling of oral rinse was 27 days (6–39 days). All ‘azole’ antifungals had higher MIC value against *Candida* isolates (irrespective of species) isolated from patients who had been previously exposed to antifungal compared with those isolated from antifungal naïve patients (fluconazole, *p* = 0.024; ketoconazole, *p* = 0.007; itraconazole, *p* = 0.004). Similarly, ‘azole’ antifungals tested were less effective against *Candida* isolates (irrespective of species) isolated from late presenters compared with those isolated from early presenters (fluconazole, *p* = 0.024; ketoconazole, *p* = 0.007; itraconazole, *p* = 0.004). No significant differences were observed (*p* > 0.005 in all cases) in MICs values of micafungin, 5-Fluorocytosine and amphotericin B between antifungal exposed and naïve patients; and between late and early presenters.

## Discussions

The present study is the first study in the era of HAART among Ethiopian AIDS patients where they present late in the course of the diseases. The study shows *C. albicans* as the predominant etiologic agent for OPC among Ethiopian late and early presenting AIDS patients with no significant difference in species distribution between the two groups of patients (*p* = 0.344). Although, studies on diversity of *Candida* species in Africa in general and in Ethiopia in particular are limited, few previous studies conducted so far demonstrated that more than 80% of oral yeast isolates from HIV-infected patients were of *C. albicans*[8,12,13,22-24] which is consistent with our findings. The distribution of *Candida* isolates from patients with recurrent OPC and/or previously exposed to antifungal drugs in the current study was different and supports the notion that repeated exposure to antifungal agents and recurrent infections might predispose to a shift to non-albicans *Candida* species [7,10] but it should be supported by further longitudinal studies. This could be due to the fact that, majority of the patients (90%) with recurrent OPC are late presenters with repeated episode of OPC and prone for repeated exposure to antifungal drugs though half of the patients had reported to a single exposure with a mean time of 27 days which may be too low to note species shitting. Indeed, three-forth of the patients with multiple *Candida* colonization had reported recurrent episodes of OPC and exposure to antifungal agents, which possibly support our hypothesis and the view that previous exposure to antifungal agents as a risk factor for *Candida* species shift. The high frequency (42%) of mixed colonization, usually *C. albicans* with other *Candida* species in our study is consistent with previous studies from HIV positive individuals [14,25] and patients with cancer [26]. The 28% isolation of non-albican *Candida* species from patients with recurrent OPC and previous exposure to antifungal agents in the current study clearly shows the pathogenic role of non-albicans species in clinical OPC among HIV/AIDS patients as it has been previously reported [25]. The presence of pseudomembraneous candidiasis and isolation of *Candida* species in 11 early presenting AIDS patients suggest that some other individual host characteristics must play a major role in determining the status of yeast carrier, which, eventually, could lead to a symptomatic infection. On top of this the low diversity of Candida species isolated from apparently health HIV negative individuals support our hypothesis and the view that previous exposure to antifungal agents as a risk factor for *Candida* species shift.

The prevalence of clinical drug resistance has increased in recent decades with the greater use and abuse of efficacious antimicrobial agents. This has been a significant problem for bacterial pathogens, where resistance to multiple antibiotics severely limits therapeutic options. In deed, antimicrobial resistance is not restricted to bacteria, however, and in the 1990s fluconazole treatment failure emerged due to the development of resistance by the fungal pathogen *C. albicans*[6]. Antifungal resistance is particularly problematic as initial diagnosis of systemic fungal infection can be delayed with the availability of limited antifungal agents and currently represents a great clinical challenge. Given the difficulties observed in the treatment of fungal infections in some groups of patients, identification of the susceptibility profile of the yeast isolate are recommended which could act as a guide in the selection and control of antifungal therapy [27]. In the current study, 32% (29/90) of the *Candida* isolates were found to be resistant to one or more antifungal agents tested which is relatively high compared with other similar studies from Africa [8,23,24] and the rest of the world [21,28]. Of the 29 Candida isolates which were resistant to one or more antifungal agents, more than half of the isolates (58%) were non albicans which may reflect the intrinsic less susceptibility of some non albicans (*C. glabrata* and *C. krusei*) to some antifungal agents like fluconazole [14]. This might be also a reflection of the inappropriate use of antifungal in the setting as the case has been observed in antibiotics use [29]. The finding of one fluconazole resistant and one SDD *C. krusei* (which is known intrinsically resistant fluconazole) among 10 isolates may indicate the influence of region-species-difference on intrinsic resistance to azole in particular and needs further investigation. Irrespective of the *Candida* species identified, 12.2% (11/90), 7.7% (7/90) and 4.7% (4) of the isolates were resistant to fluconazole, ketoconazole and itraconazole, respectively most of which are isolated from patients with recurrent episodes of OPC and previous exposure to antifungal. This indicates the presence of induction of reduced susceptibility to azoles among these groups of patients and augments on the existing data that documented patients with more recurrent episodes of OPC and repeated exposure to antifungal therapies posed a great risk of reduced susceptibility to azole antifungal agents [8,30,31]. Indeed, it has been documented that *Candida* resistance to azole compounds has frequently been attributed to a selective pressure caused by antifungal agents and azole cumulative doses due to exposure to several courses of short- or long-term suppressive therapies in patients with OPC [32].

The frequency of fluconazole resistance in literatures varies from country to country and from setting to setting in the same country. Several recent studies have reported fluconazole resistance in *Candida* strains isolated from HIV-infected patients with OPC [8,12,22,23,32,33]. In the current study taking those patients with SDD, 15% of the isolates had resistance to fluconazole which is relatively high compared with similar previous studies. This could be associated with the accessibility of the drug with free of charge with the scale up of HAART in 2005 in the country and the frequent usage of the drugs as drug of choice in the settings. The susceptibility profile of the sensitive Candida organisms (irrespective of the species) showed activity at relatively high MICs (mean MIC 7.32 μg/ml). Our study showed that, *C. albicans* generate relatively low fluconazole MICs (6.1 μg/ml) compared with *C. glabrata (*8.04 μg/ml*)* which has been reported to often generate considerably high fluconazole MICs [34]. This fluconazole MIC value is by far too high compared with a recent study from Brazil where 90% of the isolated Candida species were inhibited by fluconazole at a concentration of 2.0 μg/ml [35]. The frequency of resistance to ketoconazole and itraconazole was relatively lower and found to be 6.6% and 4.4%, respectively with MIC between 0.03–4.0 μg/ml and 0.5–8 μg/ml for ketoconzole and itraconazole, respectively which is comparable with other studies [8,28,35]. Interestingly, we found low rate of resistance with relatively low MIC value of 0.13 μg/ml for amphotericin B which in agreement with recent findings that reported most isolates of *C. albicans* were sensitive to amphotericin B [8,12,21,36] despite over 50 years of use of polyene antifungal drugs (amphotericin B) [21,36]. Our finding of concomitant resistance between fluconazole and itraconazole in 5 patients and fluconazole and ketoconzole in 2 patients agrees with the review by Rogers [37] on data from a study of *Candida* isolates in HIV-positive patients that indicates a high level of cross-resistance to itraconazole in fluconazole-resistant non albicans compared with *C. albicans* and non albicans isolates. Thus, when fluconazole resistance in *Candida* species is observed, it is generally considered preferable not to use itraconazole or other member of azoles [37].

The absence of cross-resistance between the ‘azole’ class drugs and either the polyene (amphotericin B) or echinocandins (micafungin) is in agreement with published data by Rautemaa *et al.* who reported that most isolates of *C. albicans* were sensitive to amphotericin B [36] despite the use of polyene antifungal drugs for over half century [38]. It is worse enough to note, a wide variation in MIC values of the isolates from AIDS patients and HIV negative patients in the current study. A similar finding has been reported from Brazil [21]. Two HIV-tuberculosis coinfected patients and on antituberculosis drugs for the past 2 months have reported repeat episodes of OPC and frequent exposure to various azoles and had clinical OPC for several months in spite of being infected by a susceptible strain of *C. albicans* and *C. tropicalis*. Although it is difficult to explain, it could be due to impaired drug absorption and drug interaction (rifampicin and ‘azoles’) which reduces the antifungal effect [37].

## Conclusions

HIV/AIDS patients are orally colonized by single or multiple albicans and non- albicans *Candida* species that are frequently resistant to ‘azoles’ and rarely to amphotericin B, 5-Fluorocytosine and micafungin. These could represent a serious therapeutic problem among AIDS patients with significant public health importance and highlight the need for national surveillance for examining *Candida* epidemiology and resistance to antifungal drugs. Moreover, longitudinal studies to examine the predisposing factors for non-*albicans* species colonization are also important. Producing updated information on local fungal pathogens and their sensitivity patterns is a prime tool in combating such problem. Since high rate of susceptibility was seen for polyene (amphotericin B), fluorinated pyrimidine analogue (5-Fluorocytosine) and echinocandins (micafungin), these antifungal may be used for the immediate empirical therapy of OPC for patients with recurrent episodes of OPC and in areas where culture and sensitivity testing is not available. However, the patients should be monitored regularly because of the known toxic effects of the drugs.

## Limitations

Certainly, this study have some obvious limitations such as lack of standardized procedure following CLSI protocol (including the use of non synthetic medium for susceptibility test and ATCC 102321 as quality control strains) and a lack of complete identification. The *in vitro* susceptibility of yeast to antifungal agents of drugs may depend on several factors including the growth medium used for the susceptibility testing because of possible interaction of the antifungal agent with components of the medium which may interfere with antifungal activity. Accordingly, RPMI medium is recommended for susceptibility testing of yeasts by CLSI methodology [19]. Nevertheless, it is very difficult to carry out CLSI method in developing countries like Ethiopia for various reasons. Thus, based on previously published data [20,39,40] and our observation we substituted RPMI by non synthetic medium-TSB for *in vitro* susceptibility to antifungal agents. Although, comparative studies have not been documented, the use of ATCC 10231 has been practiced and configured to produce results paralleling with the reference method and this strain was used as a quality controls strains as previously described [41]. Thus. we believe that these modifications could not result significant influence on recovery of the Candida species and susceptibility test results presented in this paper could be comparable with previous findings although minor difference are inevitable.

## Competing interests

The authors declare that they have no competing interests.

## Authors’ contributions

AM: conception of the research idea, study design, data collection and analysis and interpretation and the drafting of the manuscript; AK, BM, FM, EI: study design and analysis, interpret the data and reviewed the manuscript; BA, AG, MA, YB, FB, ZH: data collection, part of laboratory work, data analysis and reviewed the manuscript. All authors commented on the initial manuscript and approved the final manuscript.

## Pre-publication history

The pre-publication history for this paper can be accessed here:

http://www.biomedcentral.com/1471-2334/13/82/prepub
